# Market survey of disposable e-cigarette nicotine content and e-liquid volume

**DOI:** 10.1186/s12889-022-14152-2

**Published:** 2022-09-16

**Authors:** Scott Appleton, Helen Cyrus-Miller, Ryan Seltzer, Karin Gilligan, Willie McKinney

**Affiliations:** 1 Appleton Regulatory Science Services LLC, Chesterfield, VA 23838 USA; 2 HF Cyrus-Miller LLC, Sandston, VA 23150 USA; 3 Safety in Numbers LLC, Tucson, AZ 85747 USA; 4 Grey Manor Consulting LLC, Moseley, VA 23120 USA; 5 McKinney Regulatory Science Advisors LLC, Richmond, VA 23231 USA

**Keywords:** e-cigarettes, Nicotine levels, Product labeling

## Abstract

Inaccurate labels on some e-cigarette products have prompted calls for routine testing to monitor product label integrity. The objective of this study was to compare label statements of commercial disposable/non-chargeable e-cigarette products for nicotine concentration and e-liquid volume with analytically verified levels. Commercial e-cigarette samples were analyzed for nicotine concentration (*N* = 51), e-liquid volume and total nicotine content (*N* = 39). Twenty-three of the 51 samples analyzed for nicotine deviated from their label statements by more than ± 10%. Deviations ranged from -50.1% to + 13.9%. Thirty of the 39 samples analyzed for e-liquid volume deviated from their label statements by more than ± 10%. Deviations ranged from -62.1% to + 13.3%. Only one brand listed total nicotine on the label. In thirty-one of the 39 samples, calculated total nicotine amount in e-liquid deviated from the amounts calculated from the label metrics by more than ± 10%. Deviations ranged from -66.8% to -1.43%. These findings underscore the need for regulatory enforcement of manufacturing quality control and product labeling practices to optimize the harm reduction potential and consumer experience associated with the use of e-cigarette products.

## Introduction

Electronic Nicotine Delivery Systems (ENDS) have significant potential to reduce the harm associated with smoking combustible tobacco cigarettes. A 2018 report from the National Academies of Sciences, Engineering and Medicine (NASEM) concluded that e-cigarettes are far less harmful and have less dependence potential compared to tobacco burning cigarettes [1]. The report also concluded that frequent use of e-cigarettes is associated with increased likelihood of smoking cessation. More recently, following a systematic review of the relevant scientific literature, researchers concluded that more people are likely to stop smoking if they use nicotine containing e-cigarettes compared to nicotine replacement therapy (NRT), non-nicotine containing e-cigarettes, behavioral support, or no support [2]. It has been reported that a greater proportion of smokers who use ENDS with cigarette-like nicotine delivery, long term, switched completely compared to those using a placebo or cigarette substitutes [3].

The full harm reduction potential of ENDS cannot likely be achieved unless the products are labeled properly so that consumers have accurate and non-misleading information about the product and its contents. The United States Food and Drug Administration (FDA) places a high priority on assuring that consumer product labels are truthful and not false or misleading in any way [4]. For example, product labeling is an important part of FDA’s review of Premarket Tobacco Product Applications (PMTA). FDA must deny a PMTA where it finds, based on a fair evaluation of all material facts, the proposed labeling is false or misleading (Section 903 (a)(2)(B) Federal Food, Drug, and Cosmetic Act) [5].

Several market surveys of ENDS products have been conducted. In many cases, marked discrepancies between the nicotine content declared on e-cigarette product labels vs. actual measured nicotine content were reported [6–11]. Such discrepancies may reflect poor manufacturing, inadequate quality control, or losses during storage. Concerns have been expressed that inadequate or misleading product labeling could have health or dependence related implications for the consumer [10, 12]. It has also been noted that inadequate quality control and confusing or misleading product labeling could undermine consumer confidence in the integrity and efficacy of the product as a less harmful alternative to conventional cigarettes [13].

To date, most surveys of ENDS have focused on commercial e-liquid refill products. By contrast, few market surveys have examined nicotine labeling accuracy of disposable, closed system e-cigarette products. Moreover, like most packaged consumer goods, e-cigarette designs, manufacturing capability, and product labeling have evolved rapidly. ENDS manufacturers prior to FDA regulation modified their products and labeling to optimize consumer acceptance. Recent FDA decisions to deny market authorization of several e-liquids and e-cigarettes resulted in manufactures making product modifications that potentially allow continued marketing of their products (e.g., synthetic nicotine (https://time.com/6098897/vaping-companies-synthetic-nicotine/). Therefore, findings from previously published market surveys may not reflect the top ENDS brands and manufacturers.

Given market dynamics, researchers have called for routine monitoring of e-cigarette products in the marketplace [10]. Routine monitoring will most likely be a big component of any ENDS manufacturer’s post market surveillance plan. Complete and accurate information on the product label should enable consumers to reliably choose satisfying products that are a less harmful alternative to conventional cigarettes. We therefore conducted a market survey of commercial disposable/non-chargeable e-cigarette products to compare label declarations of nicotine concentration and e-liquid volume vs. analytically verified values of the same parameters.

## Materials and methods

Samples of disposable (non-chargeable) e-cigarette products purchased online or at convenience stores in the United States (US) from December 2020 to June 2021 were submitted to either Enthalpy Analytical, LLC, Labstat International Inc. or Legend Technical Services Inc. for analysis of nicotine concentration, device e-liquid volume and e-liquid density.

The nicotine concentration was determined in samples of 51 commercial disposable e-cigarette products. Each laboratory used their own in-house methods as described below. There was no statistical difference in the outcome measures among the labs, *p* > 0.05.

Enthalpy Analytical Method AM201, Version 3.1 was used for e-liquid nicotine analysis. Nicotine analysis for the e-liquid was performed via Gas Chromatography-Flame Ionization Detection (GC-FID) based on a method similar to Coresta Recommended Method No. 84 and ISO 20714. An e-liquid sample was diluted 100× in isopropanol containing the internal standard 1,4-butanediol. Separation was achieved using an Agilent 6890 Gas Chromatography oven paired with a Stabilwax 30 m x 320 µm x 1 µm column (Restek, Bellafonte, PA). Data was recorded and processed using Chromeleon (Thermo Scientific, Waltham, MA) chromatography software. The limit of detection (LOD) and the limit of quantification (LOQ) are 2.39 and 7.17 mg/g respectively for this analysis.

Labstat International analytical method, TMS-00115A, was used for e-liquid nicotine analysis. The method is based on Health Canada method ISO 20714:2019 and is applicable for mainstream Nicotine, Nicotine-Free Dry Particulate Matter, Carbon Monoxide, Humectants, Triacetin, and Menthol. For this study, only the nicotine concentration of the e-liquid was analyzed. E-liquid was mixed with an isopropanol solution containing an internal standard (trans-Anethole) using a platform shaker. The extracts were analyzed by GC-FID using a wax capillary column or equivalent. The limit of detection (LOD) and the limit of quantification (LOQ) are 0.067 and 0.224 mg/g respectively for this analysis.

Legend Technical Services analytical method, LABIND-099.3, was used for e-liquid nicotine analysis. Nicotine was analyzed on a Waters H-class Ultra Performance Liquid Chromatography (UPLC) equipped with a Waters eλ detector. The eλ detector is a Photo Diode Array (PDA). For nicotine, the 260 nm is monitored as a 2D channel for quantitation. However, the entire spectrum between 200–350 nm is acquired as a 3D channel (scan) to ensure that there are no coeluting flavorings that might interfere with the quantitation of nicotine (peak purity). Peak separation was achieved on a Waters Atlantis Premier BEH AX column with a Deionized (DI) water/methanol mobile gradient. The DI water mobile phase contained ammonium acetate and ammonium hydroxide while the methanol contained ammonium acetate. Nicotine was identified at UV wavelength of 260 nm.

E-liquid volume was measured in 39 of the 51^1^ samples using a centrifuge to extract the e-liquid from the e-liquid containing components of the devices. E-liquid density was calculated for 39 samples by dividing the mass in grams (g) of an aliquot of e-liquid by its volume (mL). We used a density of 1.12 g/mL for 11 products tested at Legend Technical Services based on published results showing similar e-liquid densities [14]. We anticipated that it was unlikely that 100% of e-liquid could be collected using the extraction method employed. We therefore assumed losses of 10% and applied an adjustment factor of plus 10% to the e-liquid volume values used in this assessment.

### Statistical analysis

Linear Mixed Models (LMM) were run to compare the difference in labeled and measured values for nicotine concentration, e-liquid volume, and total nicotine amount (nicotine concentration x e-liquid volume). LMM is used when comparing non-independent observations that may be correlated by repeated measures or other grouping factors. Each ENDS product was specified as random effect, and the brand and laboratory were entered as covariates.

Percent difference between labeled versus measured nicotine concentration was calculated by: (measured nicotine concentration - labeled nicotine concentration) / labeled nicotine concentration × 100. Difference in labeled versus measured e-liquid volume was calculated by: (measured e-liquid volume – labeled e-liquid volume) / labeled e-liquid volume × 100. Expected total nicotine amount^2^ was calculated by: (label nicotine concentration) x (label e-liquid volume). The measured total nicotine amount was calculated by: (measured nicotine concentration) x (measured e-liquid volume). Difference in expected total nicotine versus measured total nicotine amount was calculated by: (measured total nicotine amount – expected total nicotine amount) / expected total nicotine amount × 100. Differences between expected and measured percentages were categorized into a new, two-level variable of whether or not these percent differences were greater than or equal to 10%. Two other variables were also computed for whether or not the measured values were 10% greater than the labeled values and for whether or not the measured values were 10% less than the labeled values.

Chi square goodness of fit tests were run to determine if, beyond random chance, the frequency of crossing the 10% threshold was greater than zero. This method is used for one sample categorical variables to test a prespecified proportion. The test must be run with expected values greater than zero, so 0.001 was selected as the expected value for crossing the 10% threshold under the null hypothesis.

*P*-values for determining statistical significance were set at < 0.05. Statistical analyses were run with the PROC MIXED and PROC FREQ procedures in SAS^®^ Version 9.4 (SAS, Cary, NC, US).

## Results

### Percent difference of the labeled nicotine concentrations versus the measured nicotine concentrations

**Fig. 1:**
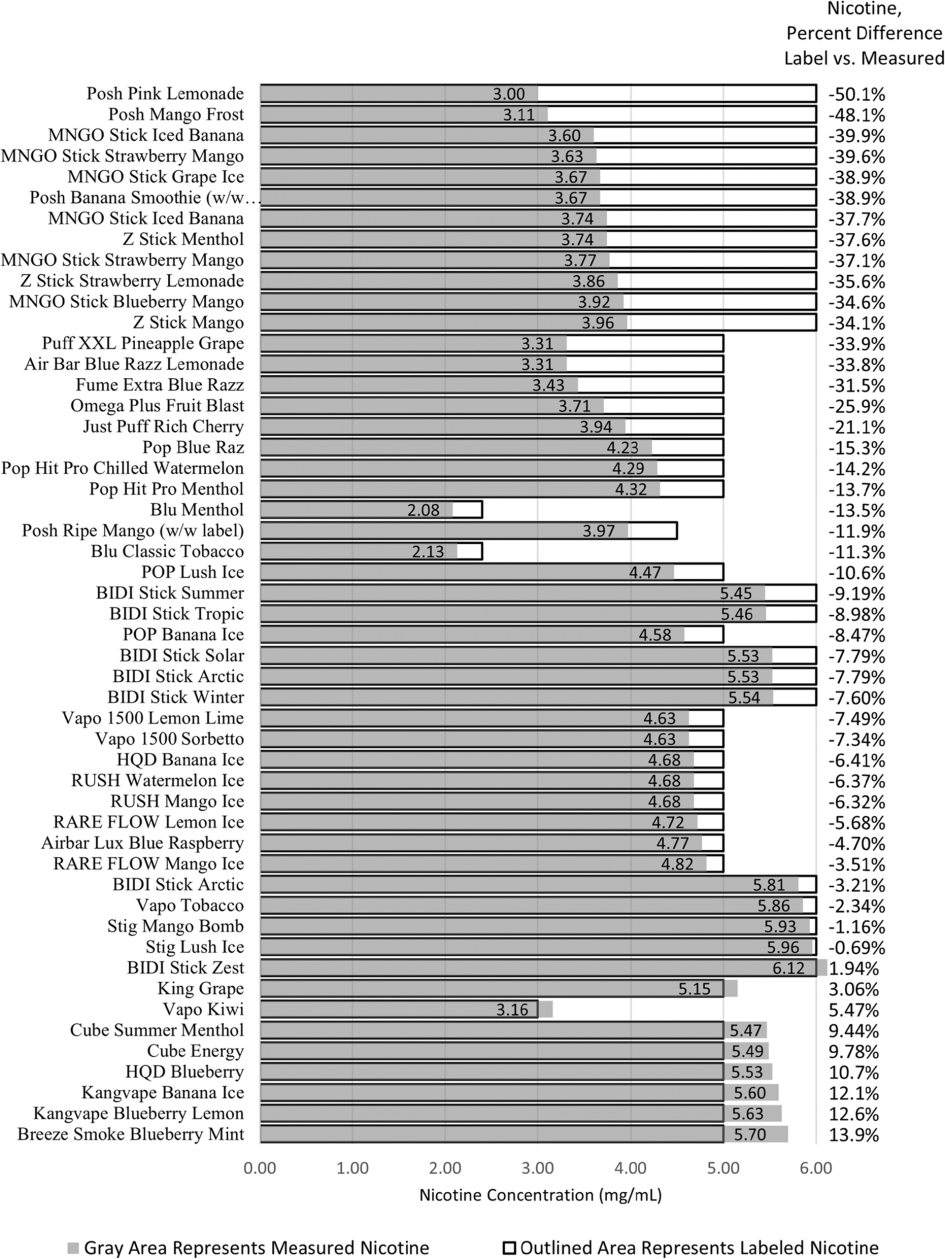
Percent Difference of Labeled vs. Measured Nicotine Concentration (*N* = 51). Comparisons of nicotine percentages using common units of mg/mL were made using an e-liquid density correction to convert nicotine mg/g to mg/mL. Density data are not shown. Measured nicotine concentrations (Grey Area) were lower than labeled nicotine concentrations (Outlined Areas) for the majority of evaluated commercial disposable/non-chargeable e-cigarette products. The percent differences from the label (Figure 1, right column) ranged from -50.1 to 13.9%

A random effects LMM showed that, when controlling for brand, the measured nicotine concentrations were significantly lower than the labeled nicotine levels, F (1, 35) = 70.94, *p* < 0.0001. Additionally, there was a significant difference between labeled and measured nicotine concentrations by brand, F (16, 35) = 10.05, *p* < 0.0001. Measured nicotine concentrations were lower than labeled nicotine concentrations for most but not all brands. Twenty four of 51 samples (47%) were less, and 4 of 51 samples (8%) were more than ± 10% of their respective label declarations. Deviations ranged from -50.07% to + 13.9%. Overall, 28 of 51 samples (55%) deviated from their label statements by more than ± 10% (Fig. 1).

The laboratory that conducted the analysis was originally included in the LMM for nicotine concentration, e-liquid volume, and total nicotine amount. This variable, however, was not significantly associated with the difference in these three outcome measures and was therefore dropped from the final models.

### Percent difference of the labeled e-liquid volume versus the measured e-liquid volume

**Fig. 2:**
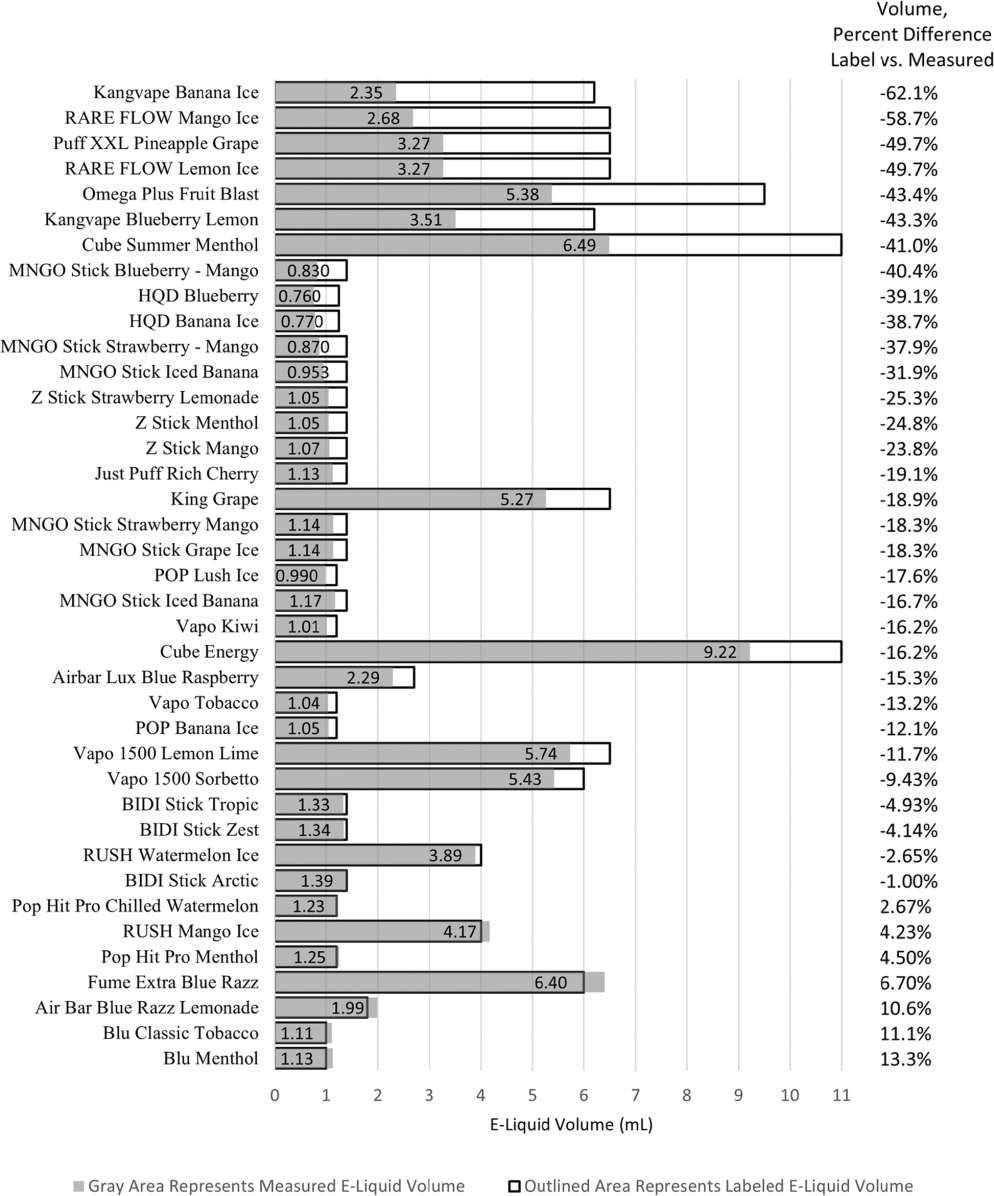
Percent Difference of Labeled vs. Measured E-liquid Volume (*N* =39). Measured e-liquid volumes (Grey Area) were lower than labeled e-liquid volumes (Outlined Area) for the majority of evaluated commercial disposable/non-rechargeable e-cigarette products. The percent differences from the label (Figure 2, right column) ranged from -62.1 to 13.3%

A random effects LMM revealed that, when controlling for brand, the measured e-liquid volume was significantly lower than the labeled e-liquid volume, F (1, 24) = 75.99, *p* < 0.0001. Additionally, there was a significant difference between labeled and measured e-liquid volume by brand, F (14, 24) = 7.57, *p* < 0.0001. Measured e-liquid volume was lower than labeled e-liquid volume for all brands. Twenty seven of 39 samples (69%) were less, and 3 samples (7.7%) were more than ± 10% of their respective label declarations. Deviations ranged from -62.1% to + 13.3%. Overall, 30 of 39 samples (77%) deviated from their label statements by more than ± 10% (Fig. 2).

### Percent difference of the expected total nicotine versus the measured total nicotine

A random effects LMM revealed that, when controlling for brand, the measured total nicotine amount was significantly lower than the expected total nicotine amount based on label information, F (1, 24) = 146.70, *p* < 0.0001. Additionally, there was a significant difference between expected and measured total nicotine amount by brand, F (14, 24) = 9.50, *p* < 0.0001. Measured total nicotine amounts were lower than expected total nicotine amounts for all brands. Thirty one of 39 samples (79%) were less, and none were more than ± 10% of their respective label declarations. Deviations ranged from -66.8% to -1.43%. Overall, 31 of 39 (79%) of samples deviated from their label statements by more than ± 10% (Table 1).

**Table 1:** Percent Difference of Expected vs. Measured Total Nicotine (*N* = 39)

**Product Name**	**Expected Total Nicotine mg**^a^	**Measured Total Nicotine mg**^b^	**Expected vs. Measured Total Nicotine Percent Difference**
Puff XXL Pineapple Grape	325	108	-66.8%
MNGO Stick Strawberry Mango^c^	84.0	31.5	-62.5%
MNGO Stick Blueberry Mango	84.0	32.7	-61.0%
RARE FLOW Mango Ice	325	129	-60.2%
MNGO Stick Iced Banana	84.0	34.3	-59.1%
Omega Plus Fruit Blast	475	199	-58.0%
Kangvape Banana Ice	310	132	-57.5%
Z Stick Menthol	84.0	39.4	-53.1%
RARE FLOW Lemon Ice	325	154	-52.5%
Z Stick Strawberry Lemonade	84.0	40.4	-51.9%
MNGO Stick Grape Ice	84.0	42.0	-50.0%
Z Stick Mango	84.0	42.2	-49.8%
MNGO Stick Strawberry Mango^c^	84.0	43.2	-48.6%
MNGO Stick Iced Banana	84.0	43.6	-48.1%
HQD Banana Ice	62.5	35.9	-42.6%
Kangvape Blueberry Lemon	310	198	-36.2%
Just Puff Rich Cherry	70.0	44.7	-36.2%
Cube Summer Menthol	550	355	-35.4%
HQD Blueberry	62.5	42.2	-32.6%
Fume Extra Blue Razz	300	219	-26.9%
Air Bar Blue Razz Lemonade	90.0	65.9	-26.7%
POP Lush Ice	60.0	44.3	-26.2%
POP Banana Ice	60.0	48.3	-19.5%
Airbar Lux Blue Raspberry	135	109	-19.2%
Vapo 1500 Lemon Lime	325	266	-18.3%
King Grape	325	272	-16.5%
Vapo 1500 Sorbetto	300	252	-16.1%
Vapo Tobacco	72.0	61.1	-15.2%
BIDI Stick Tropic	84.0	72.7	-13.5%
Pop Hit Pro Chilled Watermelon	60.0	52.9	-11.9%
Vapo Kiwi	36.0	31.8	-11.6%
Pop Hit Pro Menthol	60.0	54.1	-9.78%
RUSH Watermelon Ice	200	182	-8.85%
BIDI Stick Arctic	84.0	76.7	-8.71%
Cube Energy	550	506	-8.00%
RUSH Mango Ice	200	195	-2.36%
BIDI Stick Zest	84.0	82.1	-2.29%
Blu Menthol	24.0	23.5	-1.98%
Blu Classic Tobacco	24.0	23.7	-1.43%

^a^Expected total nicotine amount calculation: (label nicotine concentration) x (label e-liquid volume)

^b^Measured total nicotine amount calculation: (measured nicotine concentration) x (measured e-liquid volume)

^c^Samples of MNGO Stick Strawberry Mango purchased at different times were analyzed by different Labs

### Calculated percent differences with a 10% threshold

Chi square goodness of fit tests showed a significant number of cases where percent differences between measured and labeled levels of nicotine concentration, e-liquid volume, and total nicotine were above a ± 10% threshold, χ^2^ (1) = 153,684.86, *p* < 0.0001 and χ^2^ (1) = 230,732.31, *p* < 0.0001, and χ^2^ (1) = 246,372.90, *p* < 0.0001, respectively. For all these outcomes, the vast majority of ± 10% threshold difference was in the direction of measured values being lower than labeled values.

## Discussion

We conducted a market survey to compare label statements of commercial disposable e-cigarette products for nicotine concentration and e-liquid volume with analytically verified levels of the same parameters. The findings indicate large discrepancies between labeled nicotine content and e-liquid volume vs. actual measured amounts for many of the product samples evaluated in this study.

The American E-liquid Manufacturing Standards Association (AEMSA) was formed to develop standards to assure the quality and safety of e-liquid products for electronic cigarettes [15]. AEMSA guidelines state that nicotine concentrations should be within ± 10% of the concentration stated on the product label. Measured levels of nicotine and e-liquid volumes of many of the products surveyed deviated from their label statements by greater than ± 10%. In many cases, calculated total nicotine content was less than half of the expected total nicotine, indicating the consumer is getting far less than what they expected. These products could potentially be regarded as misbranded by the FDA [4].

Differences in labeled nicotine amount and measured nicotine concentrations tend to cluster by manufacturer. For example, the nicotine concentrations of MNGO Stick and Posh samples were among the lowest relative to their respective label declarations while Kangvape and Breeze Smoke products were among the highest. Eleven of 39 samples (28%) had measured total nicotine content amounts that were less than half of their expected total nicotine content amount (based on label declarations). MNGO Stick, Puff XXL, and RARE FLOW were among the samples that had the lowest measured total nicotine content relative to their respective expected total nicotine content.

Comparison of our findings with those in the published literature is complicated by the fact that our study was on e-liquids contained within closed system disposable e-cigarette devices while most published studies are on e-liquid refill products. Nevertheless, market surveys that compared label vs. measured nicotine content of e-liquid refill products frequently observe marked discrepancies between label vs. measured nicotine content consistent with our findings.

Davis and co-workers reported that 35 of 54 nicotine containing commercial e-liquid samples deviated from their product label declarations by more than ± 10%. Deviations of labeled vs. measured nicotine concentration ranged from -12.9% to + 89.7% [6]. Farsalinos and co-workers reported that 12 of 21 refill e-liquid samples had nicotine levels within ± 10% of their label statements. Overall, deviations of actual nicotine content vs. label statements ranged from − 21% to 22.1% [7]. Kim and co-workers compared labeled vs. measured nicotine content of e-liquids purchased in the United States (*n* = 32), South Korea (*n* = 29) and Poland (*n* = 30). Most of the products with inaccurate labels were those purchased in United States. Of these, 14 had discrepancies between label vs. actual nicotine content of greater than ± 10%. Overall, deviations of labeled vs. measured nicotine concentrations ranged from – 92.4% to + 103.7% [8]. Peace and co-workers measured nicotine concentrations in 27 e-liquid formulations. They reported variances between labeled vs actual nicotine concentration that ranged from -55% to + 31%. Additionally, 18 of 27 products deviated from the labeled nicotine concentration by more than 10% [9]. Lisko and coworkers compared labeled vs. measured nicotine concentration in a sample of 36 commercial e-cigarette cartridges. The investigators reported three fourths of the products contained lower nicotine levels than what was stated on the product labels [10]. Bębenek and coworkers compared labeled vs. measured nicotine and other aspects of e-liquids purchased from seven European countries. Commercially available e-cigarettes sold in Europe are only available in nicotine concentrations of 20 mg/mL or less. Only one liquid had a quantified nicotine concentration more than 10% higher than the labeled amount. Twenty-one liquids had a quantified nicotine concentration less than 10% of the labeled amount. One e-liquid with a labeled nicotine concentration of 6 mg/mL had no traces of nicotine [11].

In general, Good Manufacturing Practices (GMPs) assure proper design, monitoring, and control of manufacturing processes and facilities in order to protect public health.^3^ In the US, FDA has the authority to establish Good Manufacturing Practices or Tobacco Product Manufacturing Practices (TPMPs) for tobacco products, however, it has not yet done so. Rather than inspect for TPMP compliance, FDA inspects tobacco establishments biennially from the date of registration to ensure that they are compliant with provisions of the Food, Drug, and Cosmetic Act and that products are not adulterated or misbranded.^4^ Additional provisions FDA utilizes but are not limited to include registration and product listing; ingredient listing; requirements for packaging, labeling, and advertising; and marketing authorization for certain tobacco products.^5^ Once FDA establishes TPMPs for tobacco products, its investigators will also inspect for compliance with those requirements. Perhaps the issues identified in this publication would be addressed by the establishment of TPMPs.

A few limitations should be considered when interpreting the findings from this study. Because of the large number of e-cigarette brands currently available, the samples selected for this survey may not be representative of the overall market. Some of the products did not label e-liquid volumes. Therefore, comparisons of label information vs. actual e-liquid volumes could not be made for these samples. The labels on some products did not indicate whether the nicotine percent stated on the label was expressed as mg/g or mg/mL of e-liquid. In such cases, it we assumed the percentage was expressed as mg/g. We corrected for density to compare percentages expressed as mg/mL. This had the effect of conservatively increasing apparent nicotine concentration for products that expressed nicotine percent as mg/g. E-liquid volumes were not measured for all samples, resulting in a smaller sample size (*N* = 39 vs. 51) for the e-liquid volume and total nicotine content assessments. Although the actual amount of losses from the e-liquid extraction method is unknown, we conservatively assumed losses of 10%. However, the relative ranking of the samples remains unchanged regardless of the actual amount of e-liquid losses. The technology and regulatory oversight of these products is evolving rapidly. Therefore, this survey could be outdated in a relatively short time as manufacturers modify their products to optimize consumer acceptance and meet regulatory compliance.

A strength of this survey is that we measured e-liquid volumes in closed system, disposable e-cigarette products. Relatively few published studies have compared e-liquid volumes vs. label declarations in closed system disposable products.

Researchers have commented that variations in product characteristics such as nicotine content could impact health, safety, and dependence potential of e-cigarettes [10, 12]. As noted above, some products contained substantially less total nicotine than the expected amount based on product label. Not only is the consumer getting less than what they expect, such products may be less effective as alternatives to tobacco burning cigarettes. Results of a recently published randomized placebo-controlled trial concluded that “ENDS with nicotine delivery approaching that of a cigarette are more effective in enabling ambivalent cigarette smokers to quit smoking” [3]. Moreover, misleading labeling could erode public confidence in the benefit of ENDS as a less harmful alternative to conventional cigarettes. Label discrepancies may be caused by poor manufacturing quality control, losses during storage, or questionable business practices.

In order to optimize the harm reduction potential of ENDS, it is imperative that these products are manufactured using quality control practices, the highest-grade materials, and have product labeling that is clear, accurate, and conforms to applicable quality and regulatory standards.

## Conclusions

This study underscores the need for regulatory enforcement of manufacturing quality control and product labeling practices to optimize the harm reduction potential and consumer experience associated with the use commercial disposable/non-chargeable e-cigarette.

## Data Availability

The datasets used and/or analyzed during the current study available from the corresponding author on reasonable request.

## Footnotes

1 Legend Technical Services (LTS) only analyzed nicotine and did not measure volume. Therefore, we only have e-liquid volume measurements for 39 of the 51 samples. We included the LTS nicotine data to increase the sample size and to further evaluate the influence of laboratory variability.

2 Only one manufacturer provided Total Nicotine amount on their product label.

3 Centers for Disease Control and Prevention, Smoking and Tobacco Use. Accessed at https://www.cdc.gov/tobacco/data_statistics/fact_sheets/fast_facts/ on June 21, 2017.

4 Food, Drug, and Cosmetic Act § 905(g).

5 FDA, Letter to Tobacco Product Registered Establishments, August 16, 2011.
